# Near-infrared spectroscopy (NIRS) in functional research of prefrontal cortex

**DOI:** 10.3389/fnhum.2015.00274

**Published:** 2015-05-12

**Authors:** Nobuo Masataka, Leonid Perlovsky, Kazuo Hiraki

**Affiliations:** ^1^Primate Research Institute, Kyoto UniversityInuyama, Japan; ^2^Psychology Department, Northeastern UniversityBoston, MA, USA; ^3^Department of General Systems Studies and Center for Evolutionary Cognitive Sciences, The University of TokyoTokyo, Japan

**Keywords:** near infrared spectroscopy, prefrontal cortex (PFC), functional magnetic resonance imaging (fMRI), oxy-hemoglobin, hemodynamics, cognition, emotions

Functional near-infrared spectroscopy (fNIRS) uses specific wavelengths of light to provide measures of cerebral oxygenated and deoxygenated hemoglobin that are correlated with the functional magnetic functional imaging (fMRI) BOLD signal. fNIRS has emerged during the last decade as a promising non-invasive neuroimaging tool and has used to monitor various types of brain activities during motor and cognitive tasks with increasing interest from research communities. One can see classical comprehensive reviews of such investigations elsewhere (Hoshi, 2003; Koizumi et al., 2003; Obrig and Villringer, 2003). While various MRI methods documented in the literatures have provided essential information about the brain systems concerning these issues up to the present, they have two major limitations, their requirement for participant immobility and their high operational cost. Particularly, the former rules out the use of such techniques for investigating brain dynamics during every activities such as walking and running. On the other hand, fNIRS can be used under less body constraints than other imaging modalities require. Moreover, it is quiet (no operating sound). Thus, the conditions for performing fNIRS measurements are much more comfortable for human as well as non-human subjects (Wakita et al., 2010). In addition, it provides higher temporal resolution (faster sampling frequency). In this respect, fNIRS is useful for studying brain activity under more “natural” and therefore much more variable conditions though it can only monitor cortical regions with less spatial resolution (usually in the centimeter range).

Since fNIRS is a non-invasive, safe and portable method, topics that NIRS researches could address are highly variable, and more diverse than those with other non-invasive imaging research techniques. That fact makes fNIRS an ideal candidate for monitoring activity of prefrontal cortex, which has been undertaken with respect to more broadly based issues across the broad expanse of research communities. In fact, many academic journals in diverse areas publish findings obtained from pioneering fNIRS measurements of prefrontal regions. We must admit that this diversity of fNIRS applications makes difficult to access accumulated experience. Protocols of fNIRS recordings from various investigations are not necessarily well-standardized and not necessarily well-grounded on available knowledge of appropriate fNIRS use. One of the purposes of the current research topic is to address this issue across both theoretical and empirical aspects.

In order to realize the high potential of fNIRS, effective discrimination between physiological noise originating from forehead skin hemodynamic and cerebral signals is a pre-requisite. Main sources of physiological noise are global and local blood flow regulation processes on multiple time scales. Having attempted to identify the main physiological noise contributions in fNIRS forehead signals and to develop a method for physiological de-noising of fNIRS data, Kirilina et al. (2013) documents a set of physiological regressors, which are used for physiological de-noising of fNIRS signals. New imaging methods to fuse fNIRS measurements and fMRI data are presented to reveal the spatiotemporal dynamics of the hemodynamic responses with high spatiotemporal resolution across the brain (Yuan and Ye, 2013). Recognition algorithm is described for recognition whether one taps the left hand or the right hand (Hai et al., 2013). In the study, data with noises and artifacts collected from a multi-channel system is pre-processed using a Savitzky-Golay filter for getting more smooth data. These results are confirmed by another study (Wakita, 2014).

A more ambitious and challenging attempt is set out by two groups of scientists independently from each other, but in a similar manner, to explore the possibility that fNIRS can serve as a replacement of fMRI in clinical settings. In order to examine whether fNIRS is equipped with necessary sensitivity as a potential alternative of fMRI, they investigate the sensitivity to detecting linear changes in activation and functional connectivity in response to cognitive load, and functional connectivity changes when transitioning from a task-free resting state to a task performance (a letter n-back task with three load conditions). The results demonstrate that fNIRS is sensitive to both cognitive load and state (Herff et al., 2014). Interesting, almost identical findings are reported by another group of scientists (Fishburn et al., 2014). These findings strongly indicate the fNIRS-measured prefrontal activity to discriminate cognitive states in real life. Arguments that support this conclusion as well as suggestive evidence for the claim are presented (Derosiere et al., 2013; Harrivel et al., 2013; Yoshino et al., 2013a,b).

The most intensively investigated issue in the functional research of prefrontal cortex is that concerning working memory, particularly executive function. It is not surprising therefore that several articles deal with the issue in the current research topic. A review (Moriguchi and Hiraki, 2013) summarizes quite concisely recent advancements by fNIRS research on the development of executive function in children. It particularly concentrates on the lateral prefrontal cortex, focusing on inhibitory control and cognitive shifting. The remaining four research articles deal with divergent issues, e.g., music (Ferreri et al., 2013), story-telling (Moro et al., 2013), the Scarborough adaptation of the Tower of London (Ruocco et al., 2014), and gambling (Bembich et al., 2014). While actual issues addressed in the studies are variable, they commonly deal with encoding and decoding processed of memories stored in the working memory system for our dairy activities. This diversity of topics addresses multifaceted aspects of applying NIRS to various scientific disciplines such as pediatrics, psychology, psychiatry, neurophysiology.

Another research direction addresses emotional influences on prefrontal activity, the number of reports in this area has been increasing. In particular, processing emotional visual stimuli to produce these effects is drawing attention. An article (Doi et al., 2013) reviews unique characteristics of fNIRS as an effective tool for investigating the role of the prefrontal cortex in emotional processing. Admitting several obstacles in the application of fNIRS to emotion research, they discuss the implications of recent findings to assess the effects of prefrontal activation on emotion, specifically addressing the methodological challenges of NIRS measurements with respect to the area of emotion research. They discuss potentials of the two research fields for investigating (i) biological pre-dispositions influencing prefrontal responses to emotional stimuli and (ii) neural mechanisms underlying the bi-directional interaction between emotion and action, have much to gain from the use of fNIRS. The latter issue is also addressed by another group of scientists (Sato et al., 2014), who reports that the mood-cognition interaction occurs in the lateral prefrontal cortex, a brain region known to associate with the executive function of the working memory system closely (Moriguchi and Hiraki, 2013). Other two studies concentrate on recordings in the activation of medial prefrontal cortex when viewing visual images (non-art pictures or art pieces). In both studies, some modulation of the brain activation is confirmed. However, it is more robust when presenting art pieces (Kreplin and Fairclough, 2013) than non-art pictures (Ozawa et al., 2014). Among such art pieces, the activation was more robust when the presented images were evaluated as more esthetic than when they were evaluated as less esthetic. Based on the findings, the authors of the article conclude the activated brain region is involved during positive evaluation of visual art that may be related to judgment of pleasantness or attraction.

In summary, this research topic has provided a forum for scientists planning functional studies of prefrontal brain activation using fNIRS. We hope this will serve as a reference repository of knowledge from these fields as well as a conduit of information from leading researchers. In addition it offers an extensive cross-referencing system that will facilitate search and retrieval of information about NIRS measurements in activation studies. Researchers interested in fNIRS would benefit from an overview about its potential utilities for future research directions.

## Conflict of interest statement

The authors declare that the research was conducted in the absence of any commercial or financial relationships that could be construed as a potential conflict of interest.

## References

[B1] Bembich S. Clarici A. Vecchiet C. Baldassi G. Cont G. Demarini S. (2014). Differences in time course activation of dorsolateral prefrontal cortex associated with low or high risk choices in a gambling task. Front. Hum. Neurosci. 8:464. 10.3389/fnhum.2014.00464 25009486 PMC4067729

[B2] Derosiere G. Mandrick K. Dray G. Ward T. E. Perrey S. (2013). NIRS-measured prefrontal cortex activity in neuroergonomics: strength and weakness. Front. Hum. Neurosci. 7:583. 10.3389/fnhum.2013.00583 24065906 PMC3777133

[B3] Doi K. Nishitani S. Shinohara K. (2013). NIRS as a tool for assaying emotional function in the prefrontal cortex. Front. Hum. Neurosci. 7:770. 10.3389/fnhum.2013.00770 24302904 PMC3831266

[B4] Ferreri L. Aucoutrier J.-J. Muthalib M. Bigand E. Bugaiska A. (2013). Music improves verbal memory encoding while decreasing prefrontal cortex activity: an fNIRS study. Front. Hum. Neurosci. 7:779 10.3389/fnhum.2013.00779 PMC3857524 24339807

[B5] Fishburn F. A. Norr M. F. Medvedev A. V. Vadya C. J. (2014). Sensitivity of NIRS to cognitive state and load. Front. Hum. Neurosci. 8:76. 10.3389/fnhum.2014.00076 24600374 PMC3930096

[B6] Hai N. T. Cuong N. Q. Khoa T. Q. D. Toi V. V. (2013). Temporal hemodynamic classification of two hands tapping using functional near-infrared spectroscopy. Front. Hum. Neurosci. 7:516. 10.3389/fnhum.2013.00516 24032008 PMC3759001

[B7] Harrivel A. R. Weissman D. H. Noll D. C. Peltier S. J. (2013). Monitoring attentional state with fNIRS. Front. Hum. Neurosci. 7:861. 10.3389/fnhum.2013.00861 24379771 PMC3861695

[B8] Herff C. Heger D. Fortmann O. Hennrich J. Putze F. Schultz T. (2014). Mental workload during n-back task – quantified in the prefrontal cortex using fNIRS. Front. Hum. Neurosci. 7:935. 10.3389/fnhum.2013.00935 24474913 PMC3893598

[B9] Hoshi Y. (2003). Functional near-infrared optical imaging: utility and limitations in human brain mapping. Psychophysiology 40, 511–520. 10.1111/1469-8986.00053 14570159

[B10] Kirilina E. Yu N. Jelzow A. Wabniz H. Jacobs A. M. Tachitsidis I. (2013). Identifying and quanifying main components of physiological noise in functional near infrared spectroscopy on the prefrontal cortex. Front. Hum. Neurosci. 7:864. 10.3389/fnhum.2013.00864 24399947 PMC3865602

[B11] Koizumi H. Yamamoto T. Maki A. Yamashita Y. Sato H. Kawaguchi H.. (2003). Optical topography: practical problems and new applications. Appl. Opt. 42, 3054–3062. 10.1364/AO.42.003054 12790457

[B12] Kreplin U. Fairclough S. (2013). Activation of the rostromedial prefrontal cortex during the experience of positive emotion in the context of esthetic experience. A fNIRS study. Front. Hum. Neurosci. 7:879 10.3389/fnhum.2013.00879 PMC3868912 24391572

[B13] Moriguchi Y. Hiraki K. (2013). Prefrontal cortex and executive function in young children: a review of NIRS studies. Front. Hum. Neurosci. 7:867. 10.3389/fnhum.2013.00867 24381551 PMC3865781

[B14] Moro S. B. Cutini S. Ursini M. L. Ferrari M. Quaresima V. (2013). Prefrontal cortex activation during story encoding/retrieval: a multi-channel functional nearinfrared spectroscopy study. Front. Hum. Neurosci. 7:925 10.3389/fnhum.2013.00925 PMC3876278 24427131

[B15] Obrig H. Villringer A. (2003). Beyond the visible-imaging the human brain with light. J. Cereb. Blood Flow Metab. 23, 1–18. 10.1097/00004647-200301000-00001 12500086

[B16] Ozawa S. Matsuda G. Hiraki K. (2014). Negative emotion modulates prefrontal cortex activity during a working memory task: a NIRS study. Front. Hum. Neurosci. 8:46. 10.3389/fnhum.2014.00046 24574991 PMC3918646

[B17] Ruocco A. C. Rodrigo A. H. Lam J. Domenico S. I. D. Graves B. Ayaz H. (2014). A problem-solving task specialized for functional neuroimaging: validation of the Scarborough adaptation of the Tower of London (S-TOL) using near-infrared spectroscopy. Front. Hum. Neurosci. 8:185 10.3389/fnhum.2014.00185 PMC3975118 24734017

[B18] Sato H. Dresler T. Hauensringer F. Fallgatter A. J. Ehlis A.-C. (2014). Replication of the correlation between natural mood states and working memory-related prefrontal activity measured by near-infrared spectroscopy in a German sample. Front. Hum. Neurosci. 8:37 10.3389/fnhum.2014.00037 PMC3915104 24567710

[B19] Wakita M. (2014). Broca's area processes the hierarchial organization of observed action. Front. Hum. Neurosci. 7:937. 10.3389/fnhum.2013.00937 24478668 PMC3894456

[B20] Wakita M. Shibasaki M. Ishizuka T. Schnackenberg J. Fujiwara M. Masataka N. (2010). Measurement of neuronal activity in a macaque monkey in response to animate images using infrared spectroscopy. Front. Behav. Neurosci. 4:31. 10.3389/fnbeh.2010.00031 20676236 PMC2912168

[B21] Yoshino K. Oka N. Yamamoto K. Takahashi H. Kato T. (2013a). Functional brain imaging using near-infrared spectroscopy during actual driving on an expressway. Front. Hum. Neurosci. 7:882. 10.3389/fnhum.2013.00882 24399949 PMC3871711

[B22] Yoshino K. Oka N. Yamamoto K. Takahashi H. Kato T. (2013b). Correlation of prefrontal cortical activation with changing vehcle speeds in actual driving: a vector-based functional near-infrared spectroscopy study. Front. Hum. Neurosci. 7:895 10.3389/fnhum.2013.00895 PMC3872330 24399953

[B23] Yuan Z. Ye J. C. (2013). Fusion of fMIRS and fMRI data: identifying when and where hemodynaic signals are changing in human brains. Front. Hum. Neurosci. 7:676. 10.3389/fnhum.2013.00676 24137124 PMC3797402

